# Position of meristems and the angles of the cell division plane regulate the uniqueness of lateral organ shape

**DOI:** 10.1242/dev.199773

**Published:** 2022-12-12

**Authors:** Ayaka Kinoshita, Makiko Naito, Zining Wang, Yasuhiro Inoue, Atsushi Mochizuki, Hirokazu Tsukaya

**Affiliations:** ^1^ Graduate School of Science, The University of Tokyo, Tokyo 113-0033, Japan; ^2^ Department of Micro Engineering, Kyoto University, Kyoto 615-8540, Japan; ^3^ Institute for Life and Medical Sciences, Kyoto University, Kyoto 606-8507, Japan

**Keywords:** AN3/GIF1, Cell division, Lateral organ, Leaf meristem, Morphology, Vertex model, *Arabidopsis thaliana*

## Abstract

Leaf meristem is a cell proliferative zone present in the lateral organ primordia. In this study, we examined how cell proliferative zones in primordia of planar floral organs and polar auxin transport inhibitor (PATI)-treated leaf organs differ from those of non-treated foliage leaves of *Arabidopsis thaliana*, with a focus on the accumulation pattern of ANGUSTIFOLIA3 (AN3) protein, a key element for leaf meristem positioning. We found that PATI-induced leaf shape changes were correlated with cell division angle but not with meristem positioning/size or AN3 localisation. In contrast, different shapes between sepals and petals compared with foliage leaves were associated with both altered meristem position, due to altered *AN3* expression patterns, and different distributions of cell division angles. A numerical simulation showed that meristem position majorly affected the final shape but biased cell division angles had a minor effect. Taken together, these results suggest that the unique shapes of different lateral organs depend on the position of the meristem in the case of floral organs and cell division angles in the case of leaf organs with different auxin flow.

## Teaser

**Summary:** Different lateral organs with different morphology possess different properties of meristems; cell division angles, position of cell proliferative area and AN3 localization patterns. This study examined their roles using simulation analyses.

## INTRODUCTION

The shape of leaves plays an important role in determining their photosynthetic function (Nicotra et al., 2011). Moreover, the shape of floral organs, which are evolutionarily derived from leaves, is also important for reproductive success (Fu et al., 2022). Therefore, the shape of these lateral organs varies among species to maximize their survival ability in their natural habitats. To understand these variations, it is important to assess their developmental properties.

The centre of morphogenesis in plants is the meristem, where active cell division occurs. The shoot apical meristem (SAM) is essential to produce new above-ground organs. Regarding lateral organs, especially for leaf primordia, the leaf meristem supplies cells to the leaf blade. Thus, researchers have investigated the nature of the meristem to understand the morphogenesis of lateral organs (Esau, 1977; Poethig and Sussex, 1985; Donnelly et al., 1999; Kazama et al., 2010; Ichihashi et al., 2011).

In many angiosperms, such as *Arabidopsis thaliana*, the leaf meristem is at the base of each leaf (Tsukaya, 2014; 2021). Cell proliferation initially occurs throughout the leaf primordium but is restricted to its basal regions as the leaf primordium grows further (Donnelly et al., 1999; Kazama et al., 2010). The control of the restriction of the cell proliferation zone is not completely understood. However, *ANGUSTIFOLIA 3* [*AN3*, also known as *GRF-INTERACTING FACTOR 1* (*GIF1*)], which encodes a transcriptional coactivator that is homologous to a human synovial sarcoma translocation protein (Horiguchi et al., 2005), is considered to positively control cell proliferation in leaf primordia. The spatial distribution of AN3 at the base of leaf primordia matches the cell proliferation zone, suggesting that AN3 may act as an important determinant in positioning the leaf meristem (Horiguchi et al., 2005; Kawade et al., 2017). Expression of *AN3* in the leaf meristem is also conserved in Gesneriaceae and Gramineae species (Nelissen et al., 2015; Kinoshita et al., 2020; Tsukaya, 2021). Cell division angles (CDAs) in the *AN3*-expressing region, except for areas along the margin and vasculatures, were observed to be randomized in *A. thaliana* (Yin and Tsukaya, 2016), which may contribute to the two-dimensional expansion of the leaf lamina*.* The AN3 protein moves from cell to cell (Kawade et al., 2013), forming a gradient along the proximal-distal axis on the leaf, with the leaf base presenting the highest concentration of AN3 protein (Kawade et al., 2017) and thus being involved in the positioning of the leaf meristem. However, how *AN3* expression is restricted to the basal part of leaf primordia is still unknown (Tsukaya, 2021).

AN3 also appears to be involved in the morphogenesis of each type of floral organ (Lee et al., 2009; 2014). Petals in the *an3* mutant have a smaller number of cells and a narrower shape than those of the wild type (Lee et al., 2009), as seen in the foliage leaves, suggesting a common role for AN3 in leaf and petal primordia. However, past studies have indicated that the region of the meristematic activity in petal primordia is marginal/apical in *A. thaliana* (Dinneny et al., 2004), which is clearly different from leaf primordia. The cell proliferation is observed in the entire petal primordium at first, and then in the distal region at later stages, unlike in leaf primordia (Disch et al., 2006; Anastasiou et al., 2007). Thus, if AN3 has the same role in the positional determination of the meristematic zone in petal primordia, AN3 proteins are expected to accumulate apically and not basally in petal primordia; however, no previous studies have examined this. Marginal/apical positioning of the meristematic zone in petals may be an ancestral characteristic that is directly comparable with the apical positioning of SAM (Boyce, 2007). Although the basal positioning of the leaf meristem is common in angiosperms, marginal/apical positioning in leaf primordia is also known in some ferns and gymnosperms (Boyce, 2007; Tsukaya, 2021). Therefore, a comparison of leaf primordia with petal primordia of the same species, with special emphasis on *AN3* expression, may contribute toward understanding the roles and evolutionary history of the differences in the positioning of cell proliferation activity in the primordia of these lateral organs. The roles and expression pattern of *AN3* in sepal primordia is also of interest because sepals are the neighbouring floral organ of the petals.

As mentioned above, the cell division orientation is randomized in the leaf meristem, where AN3 is localized, except for the local regions along veins and leaf margin, where an active flow of auxin (indole-3-acetic acid, IAA) is present (Yin and Tsukaya, 2016). PIN-FORMED (PIN) genes are known to control this flow via polar auxin transport (PAT) (Verna et al., 2019; Govindaraju et al., 2020). Members of the PIN family have been extensively studied as major factors in PAT, the most famous being PIN1 (Okada et al., 1991). Mutations in many PIN genes induce defects in leaf vein patterns, with wide and bifurcated midveins and altered leaf blade shapes (Sawchuk et al., 2013; Govindaraju et al., 2020). Many PAT inhibitors (PATIs) have been used to examine the role of PAT in plant organogenesis, including 2,3,5-triiodobenzoic acid (TIBA) and N-1-naphthylphthalamic acid (NPA). Through indirect evidence, they bind to the same auxin efflux carriers to inhibit their activity (Teale and Palme, 2018). When plants are treated with PATIs, their leaves exhibit abnormal leaf venation patterns, including very thick midveins and marginal veins, similar to that of *pin1* mutants (Sieburth, 1999). In addition, the leaf shape becomes rounder and shorter than that of control plants. However, to date, no study has examined why the leaf shape is altered due to the effect of PATIs on the cell proliferation pattern in leaf primordia. To fully understand the morphogenesis process of lateral organs, it is necessary to investigate the role of the lateral organ meristem on the final organ shape and factors that affect the properties of meristems.

In this study, we chose PATI-treated leaves and planar floral organs (petals and sepals) as models of leaves with altered morphology and investigated the spatial position of the cell proliferative area, CDA and possible factors that control the properties of lateral organ meristems using AN3 as a key clue. We also performed computational analysis to clarify the causal relations between cellular behaviours and organ shapes in floral organs. We used Gaussian fitting analysis and identified differences in cell proliferation properties in two aspects – position of meristem and CDA – between sepals and petals. Moreover, we examined the factors essential for the differences in shapes using a 2D-vertex model, which is a standard mathematical framework to study morphogenesis based on cellular behaviours.

## RESULTS

### Change in the length of cell proliferation zone

**Fig. 1.**
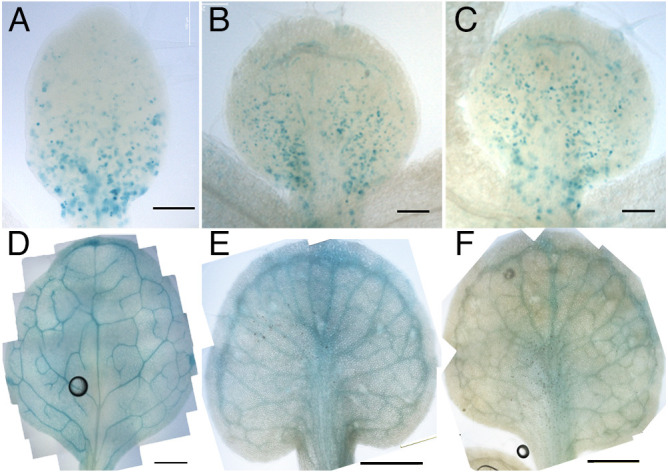
**Cell proliferation visualized by GUS staining in PATI-treated *CYCB1;1::GUS*.** (A-C) Leaf primordia from 6 DAS control (A), 7 DAS TIBA-treated (B) and 7 DAS NPA-treated (C) samples. (D-F) Leaf primordia from 12 DAS control (D), 12 DAS TIBA-treated (E) and 12 DAS NPA-treated (F) samples. Leaf primordia of similar lengths were compared. Scale bars: 100 μm (A); 200 μm (B,C); 500 μm (D-F).

**Fig. 2.**
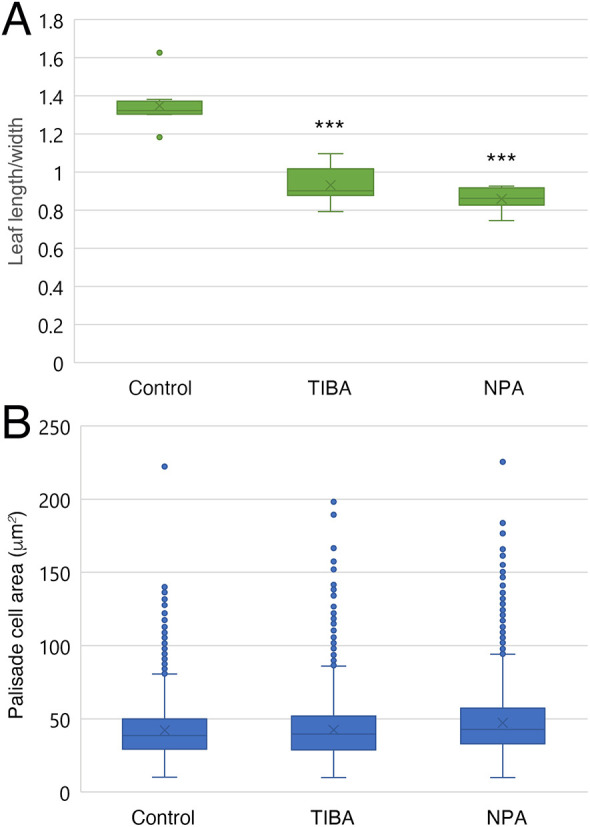
**Cellular phenotypes of PATI-treated leaves.** (A) Leaf length/width ratio of leaf primordia at cell proliferation stage. Leaf length was measured excluding the petiole. Leaf width was measured with leaves flattened out on glass slides. Left to right: 6 DAS control, 7 DAS TIBA-treated and 7 DAS NPA-treated leaf primordia are shown. *n*=4; ***P<0.001 (Dunnett's test). (B) Palisade cell area for each condition. Left to right: 6 DAS control, 7 DAS TIBA-treated and 7 DAS NPA-treated. *n*=4. Box plots show first to third interquartile ranges (boxes), 1.5× interquartile range (whiskers), median values (middle bars). The dots indicate outliers.

Firstly, we examined whether PATI treatment using TIBA or NPA affects leaf shape via changes in leaf meristem positioning in *A. thaliana.* In the PATI-treated plants, the leaves became shorter and rounder than those of the control plants (Fig. 1D-F, Fig. 2). The leaf vein pattern also differed in PATI-treated leaves (Fig. 1D-F). Namely, the midvein was widened, the lateral veins ran parallel to each other and the veins adjacent to leaf margins were also widened, indicating drastic changes in leaf organogenesis. These observations are consistent with those of previous reports (Mattsson et al., 1999; Sieburth, 1999; Sawchuk et al., 2013).

To investigate the effect of inhibition of PAT on the leaf meristem, the cell proliferation zone was observed in the leaf primordia of PATI-treated plants. The *CYCB1;1::β-glucuronidase* (*GUS*) line was used to visualize dividing G2-M phase cells (Donnelly et al., 1999). Considering the growth retardation observed in the PATI-treated plants, the first and second rosette leaves of 6 days after sowing (DAS) stage seedlings for control plants and 7 DAS seedlings for PATI-treated plants were used for this experiment.

**Fig. 3.**
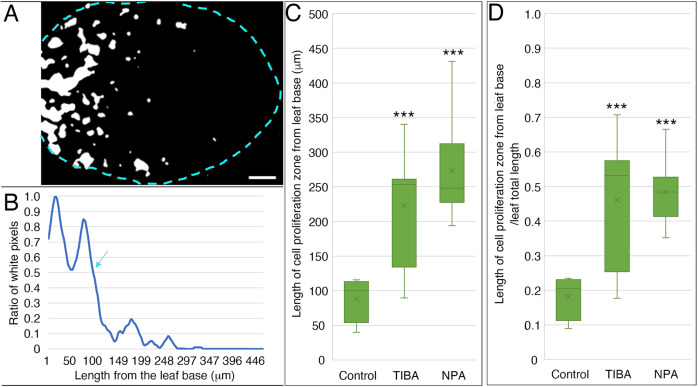
**Determination of cell proliferation zone and length and ratio of cell proliferation occupying leaf primordia.** (A) Binary images of GUS-stained leaf primordia. Scale bar: 50 μm. (B) The ratio of white pixels from binary images and length of the cell proliferation zone (>50% of white pixels) were determined. The blue arrow indicates the end of the cell proliferation zone. (C) Length of cell proliferation zone from the leaf base. (D) The ratio of cell proliferation zone to the total leaf length. *n*=8, Dunnett's test, ***P<0.001. Box plots show first to third interquartile ranges (boxes), 1.5× interquartile range (whiskers) and median values (middle bars).

In the PATI-treated plants, the cell proliferation zone remained in the proximal position, similar to the control plants (Fig. 1A-C). The images were processed to further examine the positioning of the cell proliferation zone (Fig. 3). We observed that both the length of the cell proliferation zone from the leaf base and the ratio of cell proliferation zone to the total leaf length were found to be increased in PATI-treated leaves (Fig. 3C,D).

### *AN3* mRNA and AN3 protein localization in leaf primordia

To investigate how the length of the cell proliferation zone increased, we examined the localization of *AN3* expression. As AN3 protein can move between cells, two lines, *an3-4*/*pAtAN3::AN3-GREEN FLUORESCNT PROTEIN* (*GFP*) and *an3-4*/*pAtAN3::AN3-3xGFP*, were used for the observation. The former detects actual protein localization and the latter is used to monitor accumulation of the protein without cell-to-cell movement ability, which reflects mRNA localization (Kawade et al., 2013; 2017). The first and second rosette leaves of 5 DAS seedlings were used for this analysis.

**Fig. 4.**
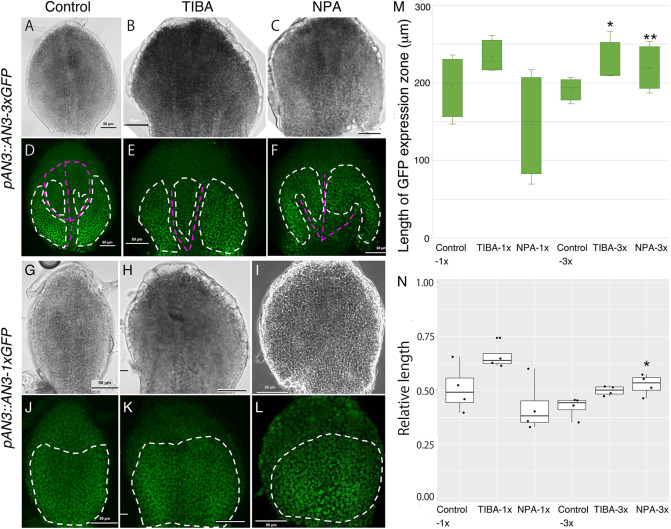
**GFP localization of *an3/pAtAN3::AN3-GFP* and *an3/pAtAN3::AN3-3xGFP.*** (A-L) Control (A,D,G,J), TIBA-treated (B,E,H,K) and NPA-treated (C,F,I,L) leaf primordia (all 5 DAS) are shown in terms of GFP localization in *an3/pAtAN3::AN3-GFP* (A-F) and *an3/pAtAN3::AN3-3xGFP* (G-L). White dotted lines indicate GFP-localized area; magenta lines indicate vasculature. Scale bars: 50 μm. (M) Length of GFP expression zone measured from leaf base. From left, *an3/pAtAN3::AN3-GFP* control, TIBA and NPA; *an3/pAtAN3::AN3-3xGFP* control, TIBA and NPA. *n*=4; *P<0.05, **P<0.001 (Dunnett's test). (N) Ratio of AN3 mRNA and protein-localized area to the total length of leaf primordia. From left, *an3/pAtAN3::AN3-GFP* control, TIBA and NPA; *an3/pAtAN3::AN3-3xGFP* control, TIBA and NPA. *n*=4; *P<0.05 (Dunnett's test). No mark implies no statistically significant difference was observed. Box plots show first to third interquartile ranges (boxes), 1.5× interquartile range (whiskers), median values (middle bars) and all sample points (dots).

We observed that the overall localization of AN3 under PATI treatment did not differ from that of the controls, remaining in the proximal region (Fig. 4A-L). This confirms the observation in the cell proliferation zone described above. The size of the *AN3*-expressing regions was also measured using the same image-processing method used for analysing the cell proliferation zone. Consequently, the *AN3*-mRNA expressing regions, monitored by the presence of the AN3-3xGFP signal, were slightly longer in the PATI-treated leaves than in control (Fig. 4M). However, AN3 protein localization of TIBA- or NPA-treated plants did not show a statistical difference from that of control plants (Fig. 4M). Therefore, irrespective of changes in *AN3* mRNA expression pattern, the spatial gradient of the AN3 protein along the longitudinal axis of leaf primordia did not change. Therefore, the influence of PATI treatment on the AN3 protein accumulation pattern is rather limited.

However, when *an3-4*/*pAtAN3::AN3-3xGFP* were treated with PATIs, we recognized that *AN3* localization was missing in the vasculature region. This missing localization was also observed in control conditions but, in PATI-treated leaves, the vasculature was very thick, and therefore, the absence became more evident (Fig. 1D-F, Fig. 4).

### CDAs in leaves

**Fig. 5.**
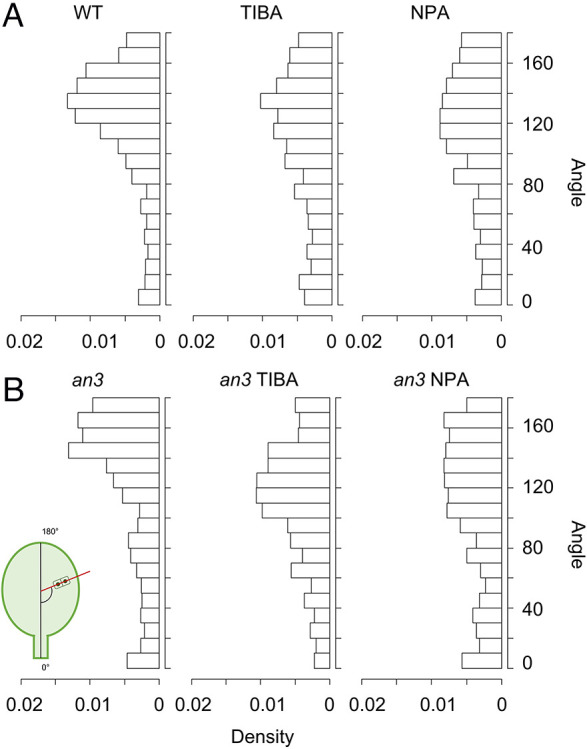
**Cell division angles in leaves.** (A) From left, wild-type (WT), TIBA and NPA; 1221, 1158 and 1061 pairs of cells were analysed, respectively. (B) From left, *an3* mutant, TIBA (*an3* mutant) and NPA (*an3* mutant); 971, 703 and 604 pairs of cells were analysed, respectively. A schematic view of the angle measurements is inserted at the lower left. Four samples were investigated for each condition.

To understand how PATI treatment caused the changes in size or pattern of the cell proliferation zone and the leaf shape, analysis of CDAs was performed using the *gl1-s92f* mutant and *gl1-s92f*/*an3-4* double mutants. We chose *gl1-s92f* mutants that lacked trichomes as a control (WT) because trichomes obstruct cell division plane observation using Aniline Blue staining (Fig. S1). The angles were determined against the proximal-distal axis, starting from the leaf base and parallel to the midvein for the first and second rosette leaves of 7 DAS seedlings (Fig. 5). As a result, a variation in CDA patterns was observed (Fig. 5A). In the control ‘WT’ plants, the CDA had a peak at approximately 130°-140° (Fig. 5A). In PATI-treated leaves, this peak was less evident, and the CDA was likely randomized (Fig. 5A). This may have contributed to changes in leaf shape, with rounder and shorter leaves, when treated with PATIs (Figs 1D-F, 2A).

We also performed a CDA investigation for the *an3-4* mutant plants. In the *an3-4* mutants without PATI treatment, the peak of CDA was around 140-150°, which differed from that of the WT (Fig. 5A,B), suggesting that AN3 might shift the CDA to smaller angles, i.e. a shift from the proximodistal to mediolateral direction. This angle shift could explain why the *an3* leaves are narrower than WT leaves. In the *an3* mutant treated with PATIs, however, the distribution of CDA became similar to that of the WT plants treated with PATIs (Fig. 5A,B), indicating that the randomizing effect of PATIs on the CDA is superior to the effect of the *an3* mutation biasing CDA to the proximodistal axis. In both WT and *an3*, the effects of PATI on the CDA appear to be similar between TIBA and NPA but slightly stronger with NPA than with TIBA (Fig. 5A,B).

### The position of cell proliferative area in floral organs

Next, we examined floral organs as modified leaves. Although floral organs such as sepals, petals, stamens and carpels are homeotic to leaves, their shapes are different. Even between sepals and petals, both of which are planar organs, there are distinct differences in shape in *A. thaliana*. For example, in the distal part, the sepal is narrower, and the petal is wider, whereas the foliage leaves are narrower in the distal part, similar to sepals.

**Fig. 6.**
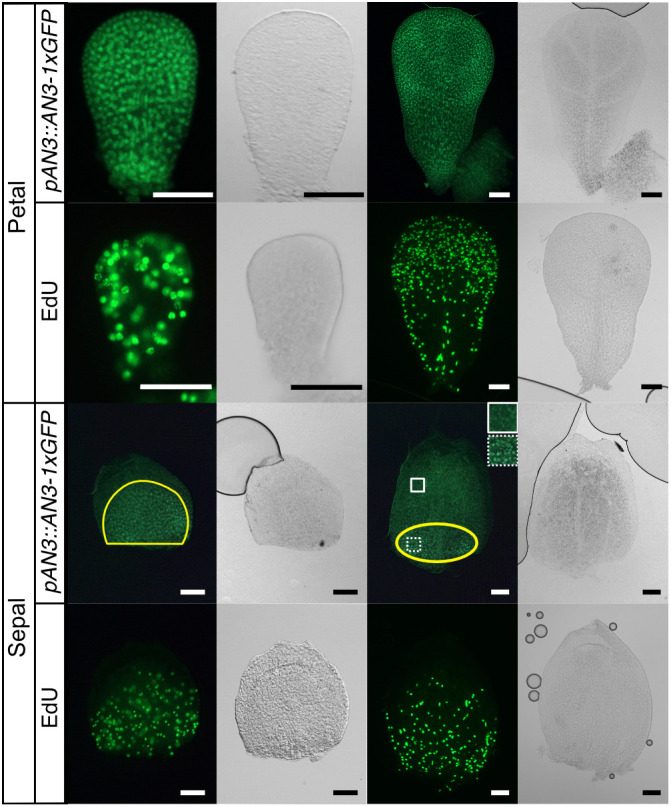
**AN3-1xGFP signals and EdU staining in sepal and petal primordia.** The primordia in the left two columns are younger than those in the right two columns. Flower stage is as follows: petal left, early stage 9; petal right, stage 10; sepal left, stage 7 (∼250 μm); sepal right, stage 8 (∼450 μm). Light microscope images are shown on the right side of each fluorescent microscope image. Images of an AN3-GFP line are in the first row of each floral organ. The second row shows the floral primordia from the EdU-treated wild type. The yellow line shows areas with brighter AN3-1xGFP signals than other parts of the primordia. Magnified views in each square of the sepal primordium are shown in the upper right. Scale bars: 50 μm.

We observed cell proliferation patterns in planar floral organ primordia to investigate whether these patterns might influence the difference in the final organ shape. To visualize dividing cells in floral organs, we used 5-ethynyl-2′-deoxyuridine (EdU), a thymidine analogue. In a sepal primordium, cell division was observed in the basal part of the organ primordium through the observed developmental stages (Fig. 6). The position of the cell proliferative area was similar to that of the leaf primordium investigated in previous studies (Donnelly et al., 1999; Kawade et al., 2017) (Fig. 1). In contrast, cell division in the petal primordia was observed in the whole organ when the organ was ∼100-150 µm in length and in the distal and marginal regions when the organ was ∼400 µm in length (Fig. 6), which marked a clear difference from that of the leaves and sepals.

### AN3 protein localization in floral organs

As AN3 is a key regulator of the leaf meristem position, we suspected that AN3 protein accumulation patterns may be different between leaves and planar floral organs, which have different positions for the cell proliferative area. Indeed, AN3-GFP signals were only observed in the basal part of sepal organ primordium, whereas the signals were observed in the entire petal organ primordium at first and then in the distal region in later stages. Moreover, in the petal primordium, sparse signals were also observed in the central region and the basal part in the later stage, stage 9 (Fig. 6), where EdU signals were rarely observed.

### AN3 protein regulates cell proliferation in sepal

**Fig. 7.**
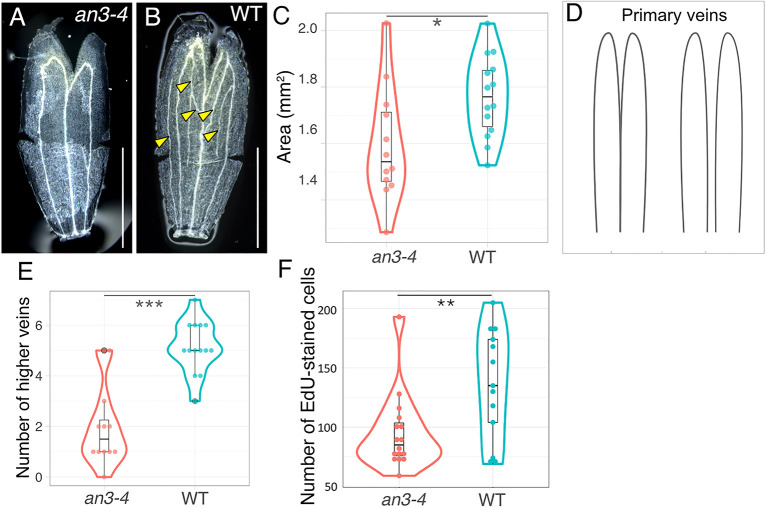
**Phenotype of the sepal in the *an3-4* and the wild type.** (A,B) Images of the sepal in each genotype: *an3-4* mutant (A) and wild type (WT; B). Some cuts were made to flatten the organs. The yellow arrowheads point to the higher veins in the sepal. Scale bars: 500 μm. (C) The area of sepals in *an3-4* (*n*=12) and WT (*n*=14). (D) Two patterns of primary veins are defined in this study. (E) The number of higher veins of the sepals in *an3-4* (*n*=11) and WT (*n*=14). (F) EdU-positive cell counts in *an3-4* (*n*=16) and WT (*n*=13). *P<0.05, **P<0.01, ***P<0.001 (unpaired *t*-test). Box plots show first to third interquartile ranges (boxes), 1.5× interquartile range (whiskers), median values (middle bars), all sample points (dots) and distribution of sample points (density curves).

It is reported that *an3* mutants have narrower petals and a smaller number of cells than the Col-0 wild type (WT) (Horiguchi et al., 2005; Lee et al., 2009); however, the sepal phenotype has not been investigated. The above-mentioned similarity in the AN3 protein accumulation pattern and proliferative area in the sepal primordia strongly suggested that *AN3* is also involved in meristematic regulation in the sepal. In order to investigate whether AN3 is involved in meristematic regulation in sepal primordia, we compared the phenotype of *an3-4* mutants and the WT in the sepals (Fig. 7A,B). The area of the organ was found to be smaller in *an3* than in the WT (Fig. 7C). We also observed that the *an3* mutant had fewer complex veins compared with the WT, which was evaluated based on the number of secondary and higher veins in the m-shaped or two n-shaped primary veins (Fig. 7D,E). To further confirm AN3 protein regulation of sepal cell proliferation, we used EdU to examine cell proliferation in the *an3-4* mutant. The cell division frequency, as quantified by EdU-positive cell count, was found to be lower in the *an3-4* mutant than in the WT (Fig. 7F). Together, these experimental data indicate that the AN3 protein regulates cell proliferation in the sepal, as is seen in the petal and foliage leaf.

### CDAs in floral organs

**Fig. 8.**
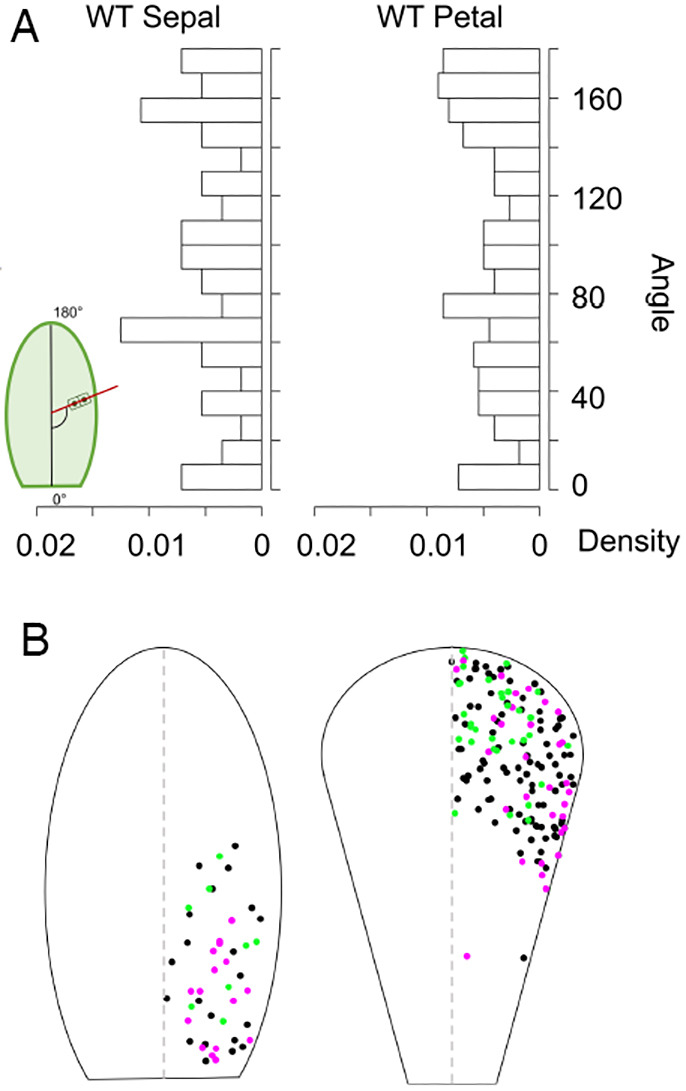
**Cell division angles in floral organs.** (A) From left, wild-type (WT) sepal and WT petal; 56 and 222 pairs of cells from four samples were analysed, respectively. A schematic view of the angle measurements is inserted in the left corner. (B) Cell division angle distribution in floral organs. Left: sepal; 46 pairs of cells from three primordia were analysed. Right: petal; 159 pairs of cells from three primordia were analysed. The angles are mapped to the right half of the diagram as the organs are symmetrical. Magenta dots indicate cell division angle in the 140°-180° range (the upper peak in panel A), green dots indicate the 60°-90° range (the lower peak in panel A), and black dots indicate angles in neither peak. *n*=3.

To know the possible contribution of biased CDA on the final shape of floral organs, CDA analysis was conducted in the sepals and petals from flowers at stages 8-10, where active cell proliferation has been identified (Alvarez-Buylla et al., 2010). The distribution pattern of CDA in planar floral organs was unique in showing loose double peaks (Fig. 8A), different from the CDA in leaves, which had a single peak (Fig. 5A,B). Irrespective of the similarity between sepals and leaves in terms of the localization of the cell proliferation zone, the overall tendency of cell division in sepals was similar to that of petals. This suggests that the pattern of CDAs is not associated with the localization of the cell proliferation zone but with organ identity.

To understand further the unique cell division patterns in these floral organs, we also analysed the spatial distribution of the CDAs in the primordia (Fig. 8B). In the sepal primordia, the cell divisions within the 60°-90° range, which were divisions in the mediolateral direction of the primordia, were identified more distally than the divisions that corresponded to the 140°-180° range, which were divisions in the proximodistal direction (Fig. 8B), and this may contribute to the oval-shaped sepal primordia. On the other hand, in the petals, the cell divisions within the 60°-90° range, which were divisions in the mediolateral direction of the primordia, were identified mostly in the central regions of the petal primordia. The divisions that corresponded to the 140°-180° range were identified mostly in the marginal regions of the petal primordia (Fig. 8B).

### Computational model of floral organ morphogenesis

Based on the above findings, we mathematically analysed the contribution of meristem position and biased CDAs on the floral organ shape using a 2D vertex model. A vertex model is a framework to calculate dynamics of cell-based morphogenesis, where each cell is modelled as a polygon and an organ is modelled as a set of polygons sharing edges between neighbouring polygons. The dynamics of organ growth are shown by movement of vertices of polygons and cell divisions (adding new vertices and edges).

In our mathematical analysis, we integrated the effect of meristem position into the model by assuming that cell division frequency is proportional to the Gaussian distribution along the proximodistal axis after a sufficient period of cell maturation, where the Gaussian parameters µ and σ indicate the peak positions (small *μ*= proximal, large *μ*= distal) and the width of the distribution, respectively. Similarly, the effect of the CDA is modelled as a bias in cell division frequency using the function β*cos 2(*θ*−*φ*), (*φ*=0, *π*/2) depending on the division angle *θ*, where *φ*=0 and *φ*=*π*/2 indicate vertically biased and horizontally biased cell division, respectively. We repeated the calculation of dynamics in 2D vertex model changing *μ* and *σ* exhaustively under different values of *β* (*β*=0 or 0.25) and *φ* (*φ*=0, *π*/2). To evaluate the distance of obtained patterns from the real floral organs (sepals or petals), we introduced a ‘difference index’, which is defined as the area of region(s) surrounded by two curves – the real and simulated organ outlines.

**Fig. 9.**
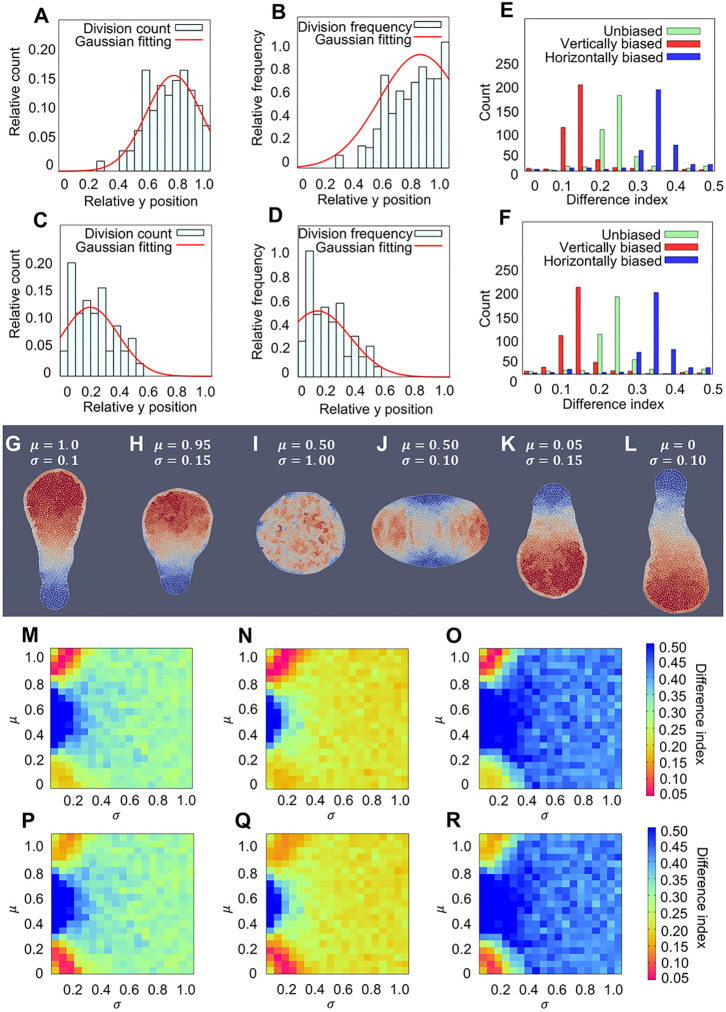
**Quantification of meristem position and floral organ simulations.** (A-D) Quantification of meristem position: (A) Gaussian fitting of petal division count (*μ*=0.76, *σ*=0.18); (B) Gaussian fitting of petal division frequency (*μ*=0.81, *σ*=0.27); (C) Gaussian fitting of sepal division count (*μ*=0.20, *σ*=0.19); (D) Gaussian fitting of sepal division frequency (*μ*=0.13, *σ*=0.22). (E,F) Histogram of difference index in sepal (E) and petal (F) obtained by exhaustive parameter search. Green boxes: unbiased division angles (*β*=0, *φ*=0); red boxes: vertically biased division angles (*β*=0.25, *φ*=0); blue boxes: horizontally biased division angles (*β*=0.25, *φ*=*π*/2). (G-L) Examples of organs generated from numerical simulations. Colour indicates division counts: red indicates higher and blue indicates lower division counts. The corresponding *σ* and *μ* values are shown in the figure. Petal difference index and sepal difference index values are: 0.206, 0.222 (G); 0.0401, 0.0154 (H); 0.346, 0.346 (I); 0.773, 0.773 (J); 0.158, 0.0717 (K); 0.219, 0.193 (L). (M-R) Heatmap of difference index on *σ*, *μ* space: random division angles (*β*=0, *φ*=0) compared with petal (M) and sepal (P); horizontally biased division angles results (*β*=0.25, *φ*=0) compared with petal (N) and sepal (Q); horizontally biased division angles results (*β*=0.25, *φ*=*π*/2) compared with petal (O) and sepal (R).

We used Gaussian fitting to quantify the cell division distribution (meristem position) on the proximodistal axis (dataset in Fig. 8). The Gaussian fitting of the cell division count and cell division frequency (count normalised by the cell number) shows highly biased division pattern on the proximodistal axis in floral organs (Fig. 9A-D). For petals, the estimated *μ* was 0.81 and *σ* was 0.19 (apical part). For sepals, the estimated *μ* was 0.13 and *σ* was 0.22 (basal part).

By numerical simulation of the model, sepal-like and petal-like shapes were obtained under some choices of parameters (Fig. 9H, petal difference index=0.0401; Fig. 9K, sepal difference index=0.0717). The green boxes in Fig. 9E,F are histograms of difference indexes for sepal and petal shapes obtained by the exhaustive parameter changes in *μ* and *σ* with *β*=0 (no bias in division angle). This shows that the successful parameter sets to simulate sepal (or petal) shape are quite limited. Fig. 9M,P are heatmaps on (*σ*, *μ*) space, where the colour indicates the difference indexes for petal and sepal shapes. They show the condition of *μ* and *σ* required for the successful morphogenesis of sepal and petal shapes. To generate the real petal shape, *σ* should be less than 0.3 and *μ* should be close to 1 (Fig. 9M). Note that too small *σ* (*σ*=0.1) and too large *μ* (*μ*=1) are not suitable to generate the real petal shape (Fig. 9G; petal difference index=0.206). Similarly, for successful generation of sepal shape, *σ* should be less than 0.3 and *μ* should be close to 0 (Fig. 9P). The optimal values of *μ* and *σ* for sepals and petals in the 2D vertex model are very close to the obtained values from measuring cell divisions in sepals and petals in real plants. These results imply that the organ shapes strongly depend on the cell division distribution (meristem position). For appropriate floral organ shapes, cell division distribution should be spatially confined, and the difference in the position of the centre of the distribution is essential to generate morphological differences between petals and sepals (Fig. 9G-L).

We also examined the condition of successful parameters under the existence of bias in the division angle (*β*=0.25, *φ*=0, *π*/2). The heatmaps (Fig. 9N,O,Q,R) show that the values of *μ* and *σ* for the successful morphogenesis of sepals (or petals) are almost the same as those under no bias in division angle (Fig. 9M,P), which implies that the division angle has little contribution to successful morphogenesis. The histograms (Fig. 9E,F) show that the biases in CDA influence the average value (or median) of the difference index. However, they do not have a major effect on changing the ratio of the successful parameter sets in the exhaustive simulations. Similar results were obtained at a larger *β* value (*β*=0.5). We also conducted an exhaustive parameter search for biased CDAs and found no real petal-like or sepal-like simulation results. In summary, these simulation results suggest that meristem position has a major effect on floral organ morphogenesis, whereas biased CDAs only have a minor effect.

## DISCUSSION

In this study, we examined how cell proliferative zones differ in primordia of PATI-treated leaves and planar floral organs from normal foliage leaf primordia of *A. thaliana*, focusing on the expression pattern of *AN3*, a key element for leaf meristem positioning (Kawade et al., 2010). We identified that PATI-induced organ shape variation cannot be attributed to changes in leaf meristem positioning or size, or the expression pattern of *AN3*, but is attributed to altered CDAs in the leaf meristem. Interestingly, the *an3* mutation biased CDA to the proximodistal axis, whereas PATI treatment randomized the angle. These effects on the CDA are reasonable considering that the *an3* has narrower leaves and PATI-treated leaves are shorter and rounder (Figs 1,2,4). Because polar auxin flow is a highly plausible reason for the polarization of the CDA (Yin and Tsukaya, 2016; Dhonukshe et al., 2005), the observed phenomena could be explained as follows: AN3 randomized the CDA (or shifted the angle from the proximodistal to mediolateral direction) against the auxin-dependent polarity; PAT inhibition randomized the CDA by cancelling the auxin flow. As *AN3* mRNA expression was not observed along the vasculatures (Fig. 1D-F), polarized CDA along the vasculature in the WT (Yin and Tsukaya, 2016) could be explained by the absence of AN3.

In addition, different shapes of sepals and petals compared with foliage leaves were found to be correlated with both altered meristem position associated with altered *AN3* spatial expression patterns and different distributions of CDAs. Hence, our results strongly suggest that lateral organ shapes are associated with differences in two aspects: position of meristem and CDAs; the former is mainly governed by the *AN3* expression pattern. Based on the mathematical simulation for planar floral organs, we also showed that the meristem position strongly influences the organ shape, whereas biased CDAs have a minor effect on the organ shape. In the following sections, several aspects of the above findings are discussed.

### The position of leaf meristem in PATI-treated plants

When *A. thaliana* plants were treated with PATIs, both the cell proliferation and *AN3-*expression zones still sat in the proximal part of the leaf primordia. It was also observed that, under the PATI treatment, the *AN3* mRNA expression zone was slightly expanded to the distal direction in the expression zone ratio, although the final AN3 protein distribution remained unchanged (Fig. 4M,N). As previously reported (Sieburth, 1999) and confirmed here (Figs 1–3), PATI-treated leaves were rounder and shorter, whereas a longer proliferation zone would be expected to produce a longer leaf if the other aspects were not changed. Instead, we found that changes in the CDAs could be attributed to the altered leaf shape.

We also observed that the *AN3* promoter was not expressed in leaf veins (Fig. 4A-L); this trend was clearly seen in PATI-treated leaves as well as in control conditions. This may happen if *AN3* expression is shut down in differentiated vascular cells, which may imply that the vasculature differentiation by auxin is superior to cell proliferation maintenance via AN3.

### CDA and leaf shape

Cell division is an important factor in both leaf development and leaf vein architecture (Kang et al., 2007). In this study, analysis of the CDA in leaf primordia revealed that the pattern differed between PATI-treated plants and control plants (Fig. 5A). This difference in the pattern could be a cause for the differences in leaf shape. In comparison with control leaf primordia that had major division angles in the 130°-140° range, PATI-treated leaf primordia had dispersed division angles in many directions, forming a round and short leaf, which matched the phenotype (Fig. 1D-F, Fig. 3). This suggests that auxin flow regulates leaf shape via controlling cell division orientation in the leaf meristem. It has been shown that the presence of vasculature is important in determining cell division patterns (Yin and Tsukaya, 2016). As auxin flow controls vascular cell polarity (Linh et al., 2018), it is possible that the cells divide in the direction of auxin flow. In addition, both NPA and TIBA affect actin dynamics (Teale and Palme, 2018; Zou et al., 2019), which can affect cytoskeletal regulation of cytokinesis, and this may be one of the underlying reasons for the change in CDA.

We observed that the *an3* mutant tended to divide at ∼140°-150°, which partially explains the narrow leaf phenotype of *an3* mutants, as cell division along the proximal-distal axis was increased (Fig. 5B). In a previous report, cell division was divided into two phases: the first has more divisions along the proximal-distal axis than the second phase (Horiguchi et al., 2011). In the latter phase, except for marginal area and local areas along veins, cell division orientation was randomized (Yin and Tsukaya, 2016). Furthermore, in *an3* mutants, the transition from the first to the second phase does not occur before the termination of cell division activity (Horiguchi et al., 2011). The shift in the peak of the CDA observed in the *an3* mutant would reflect this failure in shifting to the second phase of cell proliferation, which confirms the results of previous studies. Therefore, AN3 functions in the transition to the second phase of cell division and, consequently, cells tend to divide along the proximal-distal axis in the absence of AN3. Specifically, AN3 might promote the shift in the CDA from the proximodistal preference to the randomized one.

In addition, in *an3* mutants treated with PATIs, the CDAs were similar to those of WT treated with PATIs (Fig. 5A,B). This suggests that the randomizing effect of PATIs on the CDA is superior to that of AN3 and that the loss of PAT results in cell division in random directions irrespective of the presence or absence of AN3. This might be explained as follows: AN3 functions for the phase shift from proximodistal preference to randomized one; polar auxin transport is directly or indirectly involved in the polar-dependent CDA in both phases; loss of AN3 activity and PAT results in more proximodistal and randomized cell division, respectively. If the PAT-dependent polarity axis is lost, even in the *an3* mutant, the CDA is randomized.

### Position of meristematic tissue determines the final floral organ shape

The final organ shape could be determined by various factors, such as acceleration and deceleration of proliferation, oriented cell division and expansion, and the meristem position (Tsukaya, 2018). In this study, we showed that the position of the cell proliferative area was completely different between sepals and petals, which are both modified leaves but morphologically different. The petal of *A. thaliana*, with a modest fan shape, has a proliferative region in the distal part, which is similar to ferns with fan-shaped morphology and rare in angiosperm leaves, coinciding with a leaf meristem at the apical margin (Boyce, 2007; Tsukaya, 2014; 2018). This suggests that the morphological differences between sepals and petals could be at least partly explained by the meristematic position in each organ, which is supported by the computer simulation analysis (Fig. 9).

Although predominant cell division occurs in submarginal plate meristem to widen the leaf blade area in leaf primordia (Poethig and Sussex, 1985), it was also shown that the marginal meristem residing in the margin of the primordia is present for a short period in leaf primordia (Alvarez et al., 2016; reviewed by Tsukaya, 2021). Alvarez et al. (2016) showed that when NGATHA and CINCINNATA-class-TCP were knocked down, indeterminate marginal growth occurred in the entire margin of the leaf blade, suggesting potential meristem activity in this area. Interestingly, marginal growth occurs only in the distal region of floral organs in their system, including sepals and petals. In terms of WT petals, active cell division occurs in the distal margin in the first place (Fig. 6; Sauret-Güeto et al., 2013); therefore, the marginal meristem may contribute more than leaves or sepals. As the activity of meristem in the distal marginal area of a blade has been discussed as an ancestral character (Floyd and Bowman, 2010), petals may retain this developmental character (Boyce, 2007). We observed EdU signals in the entire margin of the petal primordia at least until the organ size was 400 µm in length, even in the proximal region. Considering the results of Alvarez et al. (2016) on leaf and floral organ primordia, the nature of proliferative cells in marginal areas is different among different lateral organs.

### Role of AN3 in planar lateral organs

Several key genes are known to positively control cell proliferation in the leaf meristem (Nakata et al., 2012; Ichihashi and Tsukaya, 2015; Tsukaya, 2021). Among them, the AN3-protein-accumulated region matches with the cell proliferative area in leaf primordia (Kawade et al., 2017), suggesting that AN3 is an important determinant of the leaf meristem position. Moreover, the smaller size of a petal in *an3* mutants or triple knockdowns of the GIF family (Kim and Kende, 2004; Horiguchi et al., 2005; Lee et al., 2009) suggested that AN3 is also involved in the promotion of cell proliferation in the petal. However, its functioning zone in primordia has not been fully investigated. In this study, we showed that the AN3-expressed region overlapped with the cell proliferative area in both sepals and petals, as observed in leaf primordia. In addition, we first showed that sepals of *an3* mutants have fewer cells as in petals or foliage leaves. These results suggest that AN3 functions as a determinant of the meristematic position and activity in all planar floral organs. However, in terms of petal primordia, AN3 protein signals were also observed in the less proliferative area. This might be due to a lack of associating transcriptional factors such as GROWTH REGULATING FACTOR5 (GRF5), which is necessary to promote cell proliferation in leaf primordia (Horiguchi et al., 2005), in such regions. Alternatively, because signals in the proximal region were not as strong as those in the distal region, the concentration of AN3 proteins might not be enough to promote cell division to the extent that EdU was incorporated.

In the past, JAGGED (JAG) was examined as a candidate regulatory gene for the specific morphology in *A. thaliana* petals that differed from leaves, because the loss-of-function *jag* mutant has narrower and shorter petals; the *JAG* overexpressor has larger petals and its mRNA is expressed in the distal margin (Sauret-Güeto et al., 2013). At that time, JAG was the only candidate ‘organizer’ that presented the petal with a pattern of growth orientations that fan out. However, AN3 has become an additional candidate that fulfils the required above-mentioned conditions. Indeed, *AN3* was identified as a direct target of JAG (Schiessl et al., 2014). The role of AN3 as the ‘organizer’ Sauret-Güeto et al. (2013) should be examined in the future.

To fully understand the mechanisms of flower organogenesis, the regulation of meristem position by floral organ identity genes needs to be investigated (Coen and Meyerowitz, 1991). Honma and Goto (2001) and Pelaz et al. (2001) revealed that when A genes (*APETALA1*) and B genes (*APETALA3* and *PISTILLATA*) were ectopically expressed together with *SEPALLATA2/3* in rosette leaves, the rosette leaves obtained petal identity, and the colour and cell shape became petal-like. However, the transformed petaloid organ was not fan-shaped but had a taper-off shape, which was similar to rosette leaves, cauline leaves and sepals. This suggests that factors other than the floral identity homeotic genes control the final organ shape. Revealing the mechanisms of *AN3* expression control might shed light on which factors are involved in the resultant different morphologies among organs.

The leaves of some gymnosperms and ferns are considered to grow from the meristem in the distal margin. The positioning of these meristems may also be determined by the spatial distribution of leaf meristem-controlling genes, such as *AN3/GIF1*. As GIF family genes exist in most eukaryotic organisms, including the basal land plants, *Marchantia polymorpha*, *Physcomitrium patens* and *Selaginella moellendorffii* (Kim and Tsukaya, 2015), further analyses of the GIFs in gymnosperms and ferns could answer this question.

### Determining the CDA in floral organs

In this study, the cell division pattern in floral organ primordia was investigated for its possible roles in the morphology of each floral organ. Both the petals and sepals showed a pattern with loose twin peaks in the distribution of CDAs, which was different from that of leaf primordia, which had a clear single peak (Fig. 6). This is an interesting new finding regarding the meristems in these primordia. In addition, we found that in the petal the cell division occurred at a 60°-90° angle in the central regions, and cell division at a 140°-180° angle was mostly in the marginal regions, whereas such a pattern was not seen in the sepals (instead longitudinal distribution was observed, Fig. 8). This difference may cause variation in shape between sepals and petals, with the distal part being wider than the proximal part, which may be caused by the cell divisions that contributed to width in the marginal regions. Although both sepal and leaf primordia have a cell proliferation zone in the basal region, the CDA was controlled differently, which may imply that CDAs depend on organ identity and affect their final shapes. Whether the randomizing effect of PATIs on CDA that was superior to that of AN3 in leaf primordia is similar for floral primordia is an interesting issue. Because inflorescence development is poor, as previously reported (Okada et al., 1991), if we cultivated plants on media supplemented with PATIs, we could not have obtained enough samples to examine in this study. To overcome this issue, we should establish a new experimental system to directly treat young floral buds with PATIs, which will be investigated in the future.

### Computational models support the importance of meristem position in organ shape control

Based on the above findings, we conducted mathematical analysis using the 2D vertex model to examine the effects of primordia position and CDA on the morphogenesis of floral organs. The numerical simulations showed sepal-like and petal-like patterns under appropriate parameter conditions. Upon exhaustive search for the parameters for quantitative evaluation of the obtained patterns, we found that the distribution of cell divisions should be strictly controlled for the successful morphogenesis of floral organs. Localization of cell divisions, which implies the existence of a meristem, is key, and the position of the meristem is essential for the differential morphogenesis between petals and sepals. In contrast to the distribution of cell divisions, the effect of CDA was minor. The horizontally or vertically biased division angle slightly influenced the conditions of successful morphogenesis of floral organs. Thus, we conclude that the meristem position largely contributes to the morphogenesis of floral organs, whereas the CDA has a minor effect.

Overall, our results imply that lateral organ shapes are likely to be regulated by two factors: the position of the cell proliferative zone governed by the spatial expression pattern of *AN3*, and CDAs. Therefore, even in one species, by changing these two factors a variety of lateral organ shapes could be achieved.

## MATERIALS AND METHODS

### Plant growth

For analysis of the leaf primordia, *A. thaliana* Col-0 (WT), or those carrying *CYCLINB1;1*(*CYCB1;1*)::GUS, *an3-4*, *an3-4*/*pAtAN3::AN3-GFP*, *an3-4*/*pAtAN3::AN3-3xGFP*, *gl-s92f* or *gl-s92f*/*an3* were grown on sterile growth medium that contained half-strength Murashige and Skoog medium (MS; Wako), 1% (w/v) sucrose (Nacalai Tesque) and 0.8% (w/v) agar (Nacalai Tesque and Wako) adjusted to pH 5.8 with potassium hydroxide. Approximately 1 M PATI (TIBA and NPA, Sigma) stocks were dissolved in dimethyl sulfoxide and added to the medium to a final concentration of 10 µM. The medium composition was based on that described by Sieburth (1999).

Seeds were sterilized by immersion in a solution of 2% (v/v) Plant Preservative Mixture^TM^ (Plant Cell Technology), 0.1% (v/v) Triton X-100 (Nacalai Tesque) and 50 mg/L MgSO_4_ for 6 h or a solution of 10% (v/v) sodium hypochlorite (Nacalai Tesque) and 1% (v/v) Triton X-100 for 5 min and twice with sterile water before plating. The plates were incubated at 24°C under constant illumination.

For analyses of the floral organ primordia, *A. thaliana* Col-0 and *an3-4*/*pAtAN3::AN3-1xGFP* (Kawade et al., 2013) were sown on rockwool (Toyobo), grown under white fluorescent light conditions (∼40 µmol m^−2^ s^−1^) at 22-23°C and supplied with water containing 1 g/l powder Hyponex (Hyponex).

### GUS experiments

Detection of GUS activity was carried out using 5-bromo-4-chloro-3-indolyl-β-D-glucuronide (X-Gluc) as a substrate. Plant tissue was first placed in 90% (v/v) acetone on ice for 10 min, washed with sodium phosphate buffer (pH 7.0) and then placed in X-Gluc buffer solution [0.5 mg/ml X-Gluc, 100 mM NaPO_4_ (pH 7.0), 5 mM K_3_Fe(CN)_6_, 5 mM K_4_Fe(CN)_6_, 10 mM EDTA, 0.1% (v/v) Triton X-100] under vacuum for 15 min or more and then placed in the dark at room temperature (∼20°C).

After GUS detection, plant tissues were rinsed in 70% (v/v) ethanol and fixed in ethanol:acetic acid (6:1) solution. After chlorophyll was removed, the tissue was preserved in 70% ethanol in the dark. Plant tissues were mounted on slides with chloral hydrate solution (50 g chloral hydrate, 5 g glycerol and 12.5 ml distilled water) (Tsuge et al., 1996) and observed under a microscope after the tissue became transparent enough.

### AN3-GFP observations

Leaf (5 DAS) and flower primordia of *A. thaliana an3-4*/*pAN3::AN3-GFP* and *an3-4*/*pAN3::AN3-3xGFP* lines were fixed in 4% (v/v) paraformaldehyde in phosphate-buffered saline (PBS) with 0.05% (v/v) Triton X-100 by immersing them in the fixation mixture, deairing them for 10 min (for leaf primordia) or 15 min (twice, for flower primordia) and placed at 4°C overnight. The samples were then washed in PBS (10 min, twice) and stored in PBS at room temperature for leaf primordia and 4°C for flower primordia until observation.

The samples were then dissected using a sharp razor under the microscope. Leaf primordia were mounted on slides with PBS and observed under a confocal microscope (FV3000; Olympus) with a GFP filter for leaf primordia and an upright fluorescent microscope (DM4000; Leica Microsystems) for floral organ primordia.

### Data analysis on the cell proliferative and AN3-GFP localized areas

We used a method derived from Kazama et al. (2010) and Ikeuchi et al. (2011) to determine the position of the leaf meristem. First, an image of a leaf with a GUS expression pattern was prepared. The outer region of the leaf was cropped, and the image was rotated so that the leaf base was on the left side of the image. Then, the blue region was extracted, and a binary image was created using ImageJ (https://imagej.nih.gov/ij/). The number of white pixels was counted for each column, and the end of the cell proliferative area along the proximal-distal axis (hereafter referred to as arrest front) was determined based on the definition of the point at which the ratio of white pixels was half that of the maximum and farthest from the blade base. The distance from the leaf base of each arrest front point was plotted for each condition in a box plot. Statistical analysis was performed using R software.

Similarly, the AN3-GFP localization area was determined as follows: first, a series of *z*-stack images were stacked using ImageJ software. Stacked images with the outer side of the leaf were cropped and rotated so that the leaf base was on the left side of the image. The region with AN3-GFP fluorescence was extracted, and from this image, a binary image was created. The number of black pixels was counted in each column. The end of the AN3-GFP localization area was determined based on the definition of the point at which the ratio of black pixels was half of that of the maximum and farthest from the blade base. The distance from the leaf base of each end of the AN3-GFP localization area was divided by the leaf length because of the size difference between lines. The obtained data were plotted for each condition in a box plot. Statistical analysis was performed using R software.

### Observation of Aniline Blue signal

We used a method derived from previous studies for the detection of newly formed cell walls (Kuwabara and Nagata, 2006; Kuwabara et al., 2011). Leaf primordia (7 DAS) of *A. thaliana glabra1(gl1)-s92f* and *gl1-s92f*/*an3-4* mutants and Col-0 flower petals and sepals were first fixed in a mixture of ethanol and acetic acid (4:1, v/v) for 30 min and then rinsed in 100% ethanol. Then, the samples were immersed in a mixture of ethanol and 100 mM phosphate buffer (pH 9.0; 1:1, v/v) for 30 min and then in 100 mM phosphate buffer (pH 9.0) for 10 min. Finally, the samples were immersed in a 0.02% (w/v) solution of Aniline Blue in 100 mM phosphate buffer (pH 9.0) for at least 7 days and up to 30 days at 4°C. Leaf primordia were mounted on slides with the staining solution and observed under a confocal microscope (FV10C-PSU; Olympus) under UV excitation with a DAPI (4′,6-diamidino-2-phenylindole) filter. The data were analysed by taking each angle of the septum wall. Calculations were performed using Microsoft^®^ Excel.

### Observation of EdU-marked cells

We used the Click-iT EdU Alexa Fluor 488 Imaging Kit (Thermo Fisher Scientific) to visualize the cells in S phase. We dissected the inflorescence of *A. thaliana* into several pieces and soaked the flower clusters in 10 µM EdU solution in half-strength MS medium with 1% sucrose for 3 h under ∼45 µmol m^−2^ s^−1^ white fluorescent light. The samples were washed with PBS containing 0.1% Triton X-100 and fixed with FAA [37% (v/v) formaldehyde, 5% (v/v) acetic acid, 50% (v/v) ethanol] and stored at 4°C. Subsequent fluorescent labelling with Alexa Fluor 488 or 555 (Thermo Fisher Scientific) was conducted according to the manufacturer's instructions. Floral organs were mounted on slides, and fluorescent dye signals conjugated to EdU were observed under a fluorescent microscope (DM4000; Leica Microsystems).

### Vertex model

To simulate the dynamics of organ morphogenesis based on cell behaviours, we used a 2D vertex model (Nagai and Honda, 2001). In the model, a plant cell is defined as a polygon and a plant organ is represented by a set of polygons sharing edges between the adjacent pair of polygons. The deformation of cells is represented by the movement of the vertices that is defined by a set of ordinary differential equations for the position of vertices. Let vector ***r***_*i*_ denote the position of *i*-th vertex. Each vertex moves toward the direction that allows a decrease in the potential energy *U* as defined below (Hayakawa et al., 2016):

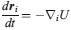


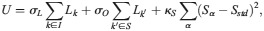
where *L*_*k*_ is the length of edge *k*, 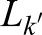 is the length of edge *k*^′^ at the tissue surface, *S*_*α*_ is the area of the cell *α*, and *S*_*std*_ is the standard cell area. *I* and *S* indicate the set of vertices inside the organ and at the surface of the organ, respectively. *σ*_*L*_ and *σ*_*O*_ denote the interface energy per unit length between two neighbouring cells and at the tissue surface, respectively. *κ*_*S*_ is the elastic constant. Parameter values were set as follows: *σ*_*L*_=0.5, *σ*_*O*_ =2.0, *κ*_*S*_ =1.0, and *S*_*std*_=3.5. Cell division occurs through generating a new line that divides the cell area by half and adding the two cross points as the two new vertices. The angle of this line is defined as the division angle, and it is either randomly distributed or controlled by biased CDAs. We started from a circular shape of 61 cells and ended the simulation upon reaching 1000 cells. The vertex model results were visualized using Paraview. All coding related to the vertex model simulation and analysis was performed using C++ codes.

### Cell division frequency and angle

For each cell, cell division in our vertex model is controlled by a value called cell time (*t*_*cell*,*i*_). During the initiation of simulation, each cell is given a random number between 0 and a value called cell time threshold (*t*_*th*_). For every 1000 numerical simulation steps, the cell time of each cell would increase by 1, and for every 10,000 numerical simulation steps, cells with cell time above the threshold (*t*_*cell*,*i*_>*t*_*th*_) would have a one in four chance to divide. In our simulation, the cell time threshold of each cell was multiplied by two modifiers – a cell area modifier and a Gaussian modifier. The area modifier is a traditional method used in vertex modelling and assures that cells with a small area will not divide and cells with large area have a better probability of division. The Gaussian modifier is defined as follows:

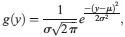
where *y* is the relative *y*-axis position of the cell. *µ* and *σ* are the normal Gaussian parameters that describe the peak position and peak width of cell division distribution, respectively. We conducted an exhaustive search of (*μ*, *σ*) parameter space for the sepal-like and petal-like simulated organ shape.

The distribution of CDAs was determined by probability density function:

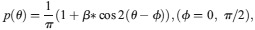
where *β* is the degree of angle bias in division and *φ* is the direction of the bias. A non-zero value of *β* means existence of angle bias. We used *β*=0 for the case of no angle bias and *β*=0.25 for biased division angle in our simulation. In case of biased cell division, we used *φ*=0 for vertically polarized cell division and *φ*=*π*/2 for horizontally polarized cell division. The generation of specific division angles for the cell division was realised using the acceptance-rejection method.

### Cell division frequency analysis and Gaussian fitting

From the Aniline Blue staining experiment, we retrieved the cell division count data in petals and sepals. In order to compare these experimental data with our simulated data, we defined cell division frequency as a function of the position along the proximal-distal axis and calculated it by dividing the cell division count by the width of the organ at each position. Then, we used Gaussian fitting to quantify the cell division count distribution and cell division frequency distribution. Gaussian fitting was performed using homemade C++ codes based on Guo's algorithm (Guo, 2011).

### Difference index

The difference index (DI) is defined as the total area of regions surrounded by real organ outline and simulated organ outline. We normalised the size of the real organ and that of the simulated organ such that their vertical (proximodistal direction) length was 1 and set 101 measuring points on both (left and right) sides of the outlines of each organ with an even distance of 0.01 in the vertical direction. Then, we measured the distance (*xd*_*i*_) in the horizontal direction between the outlines of real and simulated organs. We summed up the horizontal distance and multiplied it with the y interval and obtained:

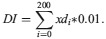


The histogram and heatmap of the DI of the simulated organ (random and biased division angles) and real organ (petals and sepals) were constructed using gnuplot. Through observation, we believe that DI<0.05 shows a high similarity between the simulated and real organ shapes.

## Supplementary Material

10.1242/develop.199773_sup1 Supplementary information
