# Supplementary material for: Position of meristems and the angles of the cell division plane regulate the uniqueness of lateral organ shape
Source: Development. 2022 Dec 12;149(23):dev199773. doi: 10.1242/dev.199773 (PMC10112895; doi:10.1242/dev.199773)
Supplement: Supplementary information [file develop-149-199773-s1.pdf]

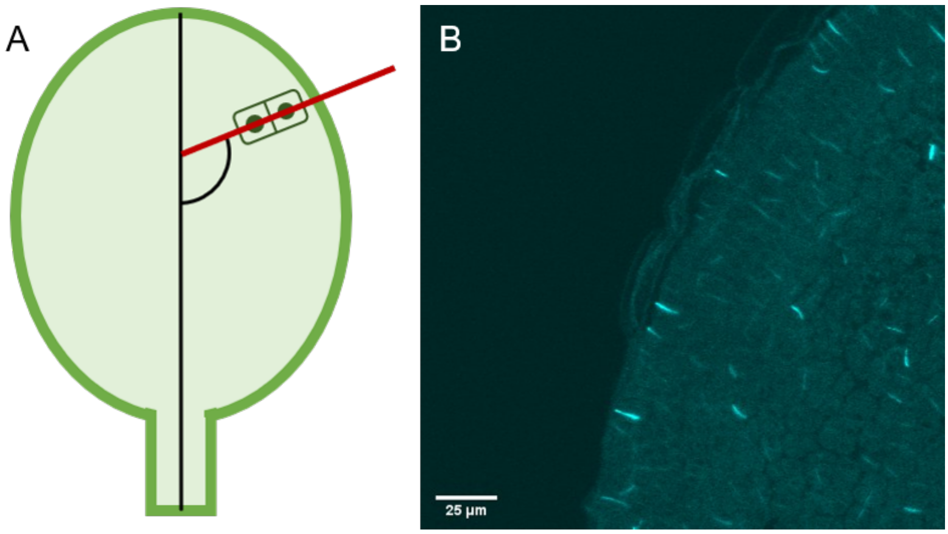

Fig. S1. The method of determining cell division angles. The angles were taken against the proximal-distal axis.

A: Schematic view of the method.

B: Picture of aniline blue-stained sample. Scale bar: 25 µm

Development • Supplementary information
